# Editorial: Particles and Health 2021: An international conference addressing issues in science and regulation

**DOI:** 10.3389/fpubh.2022.1062221

**Published:** 2022-12-05

**Authors:** Robert J. McCunney, Paul Borm, Kevin Driscoll, Nils Krueger, Len Levy

**Affiliations:** ^1^Brigham and Women's Hospital, Pulmonary Division and Harvard Medical School, Boston, MA, United States; ^2^Heinrich Heine University of Düsseldorf, Düsseldorf, Germany; ^3^Rutgers, The State University of New Jersey—Busch Campus, Piscataway, NJ, United States; ^4^Evonik Industries, Darmstadt, Germany; ^5^Cranfield University, Cranfield, United Kingdom

**Keywords:** particles, lung cancer, regulations, carbon black, amorphous silica, titanium dioxide, nanotechnology, microplastics

Regulatory initiatives in the European Union (EU) have led to proposed carcinogenicity classifications for poorly soluble low toxicity particles (PSLTs). Although PSLTs lack a widely accepted definition, commonly cited examples include carbon black, titanium dioxide and iron oxide, among others. In light of the demanding time challenges in proper review and regulation of substances used in commerce, it can be appealing to consider classes of compounds to facilitate regulatory applications. In a similar vein, the German MAK Commission has proposed workplace exposure limits for poorly soluble low toxicity particles. To foster an update on the science regarding PSLTs, and potential regulatory applications, the Particles and Health 2021 conference was designed to update the interdisciplinary science of PSLTs related to toxicology, epidemiology, occupational, and pulmonary medicine and exposure assessment, among others. This special issue focusing on “Particles and Health” includes peer reviewed papers on the topics of human health, the role of rat inhalation studies and inflammatory responses in addressing health risks, followed by papers on non-pulmonary effects of PSLTs and papers related to the regulatory impact of scientific studies related to PSLTs.

## Introduction

Regulatory initiatives in the European Union (EU) have led to proposed carcinogenicity classifications for poorly soluble low toxicity particles (PSLTs). Although PSLTs lack a widely accepted definition, commonly cited examples include carbon black, titanium dioxide and iron oxide, among others. In light of the demanding time challenges in proper review and regulation of substances used in commerce, it can be appealing to consider classes of compounds to facilitate regulatory applications. In a similar vein, the German MAK Commission has also proposed workplace exposure limits for poorly soluble low toxicity particles.

To foster an update on the science regarding PSLTs, and its potential regulatory application, the Particles and Health 2021 conference was designed to update the interdisciplinary science of PSLTs in toxicology, epidemiology, occupational and pulmonary medicine, and exposure assessment, among others (see conference web site www.particlesandhealth.org for more detail). Occupational exposure to PSLTs can occur in many industrial sectors, including mineral mining and milling; welding and asphalt use; and in the manufacture of textiles, glassware, roofing, pulp, and paper products. Nanomaterial manufacturing and use (e.g., gold, copper, titanium dioxide) presents other challenges. PSLT exposures may impact millions of workers globally.

The scientific program committee strove to select disparate perspectives on similar topics to promote discussion for the support of evidence-based scientific underpinning of regulatory standard setting regarding PSLTs. Although regulatory concern regarding PSLTs has primarily focused on lung cancer risk based on rat inhalation overload studies, the conference included presentations on genetic, reproductive and cardiac issues, among others.

The conference goals were to present current scientific information on particles and health regarding risks to human health and the environment while specifically addressing: (1) Uncertainties of defining poorly soluble low toxicity particles (PSLTs); (2) Whether PSLTs should be considered separate entities or broadly defined; and (3) Appropriate regulatory applications of the health related science of PSLTs.

The conference was divided into four major sections held over a two period in which 30 formal presentations were made, including:

1. The role of human studies, including epidemiology, in assessing health risk.
2. The role of animal and *in vitro* studies in assessing inflammation as a key adverse outcome pathway in particle induced effects.
3. The role of nanoparticle toxicology and potential impact of PSLTs on non-pulmonary adverse effects.
4. The critical aspect of appropriate regulatory application of science.

What follows are highlights of the key sections led by the respective moderators of the session.

## Human studies

Role of human studies in assessing health risk.

The Health section was introduced by a discussion of the history of The Institute of Occupational Medicine (IOM) from its inceptions in the 1960s when it played a major investigative role in understanding coal workers pneumonoconiosis to today's challenges in addressing the potential impact of nanomaterials (Seaton et al.).

In the regulatory application of scientific studies regarding health implications of the production and use of certain materials, including PSLTs, human studies led by Mundt et al., most notably epidemiological evaluations have particular relevance. In fact, according to CLP regulation (EC) No 1272/2008 (Annex I, Section 1.1.1.4), complementing the European REACH Regulation (EC No 1907/2006), “*Where evidence is available from both humans and animals and there is a conflict between the findings, the quality, and reliability of the evidence from both sources shall be evaluated to resolve the question of classification. Generally, adequate, reliable, and representative data on humans (including epidemiological studies, scientifically valid case studies as specified in this Annex or statistically backed experience) shall have precedence over other data.”*

The US Centers for Disease Control has also noted that Epidemiology is the preferred basis of Risk Evaluation. Epidemiology, often described as the basic science of public health, can demonstrate human health risks of PSLTs under the “natural” circumstances of use and exposure. In a presentation regarding the “Role of epidemiology in human risk assessment,” Mundt et al. reported on several chronic inhalation studies of PSLTs (e.g., carbon black, TiO2, and talc) in rats that demonstrated risk of lung cancer, but only at “lung particle overload” doses in rats.

Similar findings were not observed in mice, hamsters, and guinea pigs. In contrast, epidemiological studies of talc, carbon black, and titanium dioxide workers have not shown statistically significant associations between PSLT particles and risk of lung cancer (Mundt et al.).

The value of meta-analysis in the evaluation of disparate results in individual studies was addressed by Yong et al. based on a study related to Carbon Black and Lung Cancer Mortality-A Meta-regression Analysis Based on Three Occupational Cohort Studies. Evidence of lung cancer among carbon black production workers was inconsistent: increased lung cancer mortality was indicated in the UK and the German cohorts, while deficit was found in the US cohort (1–3). Lack of exposure- response analyses were identified to be a gap by the IARC working group (4). An updated follow-up study of the US cohort was published in 2015, to address the exposure-response relationship (3). Based on a sensitivity analysis of cumulative exposure-response estimates the relative risk of lung cancer was 0.99 [95% CI: 0.994–1.005; (5)].

To address whether coal worker mortality studies can offer a perspective in addressing the significance of rat inhalation studies for human risk assessment, McCunney and Yong reviewed the coal worker cohort mortality studies and evaluated whether components of coal warrant its classification as a representative PSLT.

According to the International Agency for Research on Cancer (IARC), coal is a complex mixture of >50 elements and their oxides. Coal dust is composed of numerous substances, including Human Carcinogens, such as crystalline silica, beryllium and cadmium. Moreover, coal mining activities often occur in the context of exposure to machinery that emits diesel exhaust particles—an IARC Type I carcinogen. In contrast to coal, Carbon Black (CAS No. 1333-86-4)—a manufactured product considered a PSLT- is virtually pure elemental carbon (upwards of 98–99%) produced by incomplete combustion of gaseous or liquid hydrocarbons under controlled conditions. Its physical appearance is that of a black, finely divided pellet or powder.

Risk of lung cancer among coal miners has been investigated in cohort mortality studies conducted over nearly 50 years. Over 120,000 coal miners have been part of studies in UK, Germany, Netherlands, USA, Poland, Japan, Czech Republic, and Australia. The weight of epidemiological evidence suggests no increase in risk of lung cancer among coal miners. Slight elevations in SMR cannot lead to a reliable conclusion about an increased risk due to limitations in exposure assessment, and inherent biases in case-control studies, most notably confounding and recall bias.

Despite the lack of scientific appropriateness of using coal as a surrogate for PSLTs in evaluating the significance of rat inhalation studies, the preponderance of epidemiological results of cohort mortality studies of coal-mine workers do not indicate a consistent increase in lung cancer risk.

Since poorly soluble low toxicity particles such as carbon black or titanium dioxide have raised concern about potential malignant or non-malignant adverse effects such as lung cancer or chronic airflow obstruction, Harber summarized the application of causal inference analysis to questions concerning the health effects of such particles. Relationships with malignancies remain uncertain. Inflammation has been postulated as a key intermediary step in the pathogenesis. Observational predictive epidemiologic studies have limited ability to address poorly observable mechanistic steps. Causal inference analysis, such as the use of Directed Acyclic Graphs (DAG), can create a useful analytic framework to allow integration of data from epidemiologic, clinical, and experimental studies to address mechanistic questions. In addition, these methods are useful to clarify potential bona fide and artifactual observed relationships.

As a means of detecting early signs of pulmonary inflammation that may presage malignant and non-malignant lung disease, Professor Chris Fanta summarized the potential utility of measurement of exhaled nitric oxide. Exhaled nitric oxide assessment is a non-invasive, simple, and safe method of measuring airway inflammation that provides a complementary tool to other ways of assessing airways disease, including asthma (6). This parameter is currently used in clinical settings in the diagnosis of asthma, detection of eosinophilic inflammation of the airways, prediction of steroid responsiveness in asthma, monitoring for asthma activity and assessing medication non-adherence (7). Analysis of components of exhaled breath offers a non-invasive assessment of airway and lung pathology. The science of exhaled breath analysis, however, is in its infancy but nonetheless has potential for enhancing the understanding of the lung's inflammatory response to inhaled particles.

Considerable overlap in results may be present, however, between normal and disease and may be Influenced by age, atopy, sinus disease, and cigarette smoking, among others. Dramatic suppression by inhaled corticosteroids, for example, can occur and lead to results in a “gray zone” (20–40 ppb).

Yong et al. describe their evaluation of the effect of cumulative exposure to respirable synthetic amorphous silica (SAS) dust on respiratory morbidity, as assessed by spirometry. Multiple exposure assessments were conducted in a cross sectional analysis of 462 exposed male workers. An averaged cumulative respirable SAS dust concentration of 6.44 mg/m^3^ years was estimated. Internal regression models suggested a reduction of 8.11 ml (95% CI: 0.49–15.73) in forced vital capacity (FVC) per 1 mg/m^3^ year increase of exposure. No effect on forced expiratory volume in 1 s (FEV1) or the FEV1/FVC ratio was observed in association with exposure to the respirable fraction of SAS. No adverse effects on the occurrence of respiratory diseases were observed. The authors concluded that there was no clear evidence of adverse pulmonary effects from occupational exposure to respirable SAS.

## Rat inhalation studies

In a luncheon address, Dr. Paul Brandt-Rauf, the editor of the Journal of Occupational and Environmental Medicine, described some of the vagaries of journal publications. He described the role of the impact factor in raising a journal's reputation and reviewed some of methods employed by editors to raise a journal's impact factor, most notably by publishing major review articles, which customarily receive more citations than individual articles. The afternoon session, moderated by Borm et al. focused on inflammation as a key adverse outcome pathway in particle induced adverse health effects. Dominique Lisone presented a study related to the role of pulmonary macrophages in inflammation and the development of lung cancer. Driscoll then presented paper on particle induced inflammation and lung cancer based on the outcome of an Edinburgh workshop. Roger Duffin then presented an overview of inflammatory pathways in humans followed by a discussion by Jack Harkema, a pathologist who discussed pulmonary cell proliferation, the missing link in particle induced lung cancer. The session concluded with a group discussion related to the Global Harmonization System (GHS) based target organ toxicity design and interpretation of existing studies.

## Nanotechnology and non-pulmonary effects

Nanoparticles can be defined as particulate materials having at least one external dimension smaller than 100 nm. Airborne nanoparticles can occur naturally (silicates, iron oxides); be anthropogenic, arising as by-products of human activity, e.g., fossil fuel combustion; or can be materials purposely engineered to be of nanometer size and possessing unique properties.

The potential of inhaled nanosized particulate to cause adverse pulmonary effects is well-documented; however, their potential to have effects outside the lung is less well-understood. Epidemiology studies have demonstrated associations between inhaled ambient fine particles which includes a nanosized fraction and adverse cardiovascular effects (8, 9). More recently, other non-pulmonary responses have been attributed to inhaled nanoparticles (Scarcello et al.) (10, 11). The session covered several topics including: dose metrics and biokinetics relevant to nanoparticles; the potential for nanoparticle exposure to elicit adverse effects on DNA, the nervous system, the reproductive system and developmental toxicity, the cardiovascular system, and strategies for evaluating the safety of new nanotechnologies.

Drs. Oberdörster, Creutzenberg, and Graham discussed dosimetric and biokinetic considerations of nanoparticles. Important factors differentiating nano from larger micron sized inhaled particles include: the larger number and surface area per unit mass (or volume) of nanoparticles and consequently the potential for greater reactivity; the role of diffusion mechanisms in the deposition of nanosized materials resulting in deposition patterns in the respiratory tract which differ from larger size particles for which inertial mechanism are more important; and the nanosize potentially enabling translocation into cells and along axons and dendrites.

Regarding translocation of nanoparticles, Creutzenberg et al. summarized studies in rats with inhaled and/or intratracheally instilled titanium dioxide, zinc oxide, silica, and carbon black which demonstrated that shortly after exposure deposited nanoparticles were found as aggregates localized in macrophages. Tissue analysis after days or weeks demonstrated retention in lung macrophages, pneumocytes, and translocation to the lung associated lymph nodes. These studies supported the major clearance pathway was *via* the gastrointestinal track with no significant translocation to remote organs.

Dr. Graham described studies conducted studies on human autopsy tissues using high resolution transmission electron microscopy combined with elemental mapping which demonstrated the present of nanosized particles in the olfactory bulbs. The particle composition (e.g., silicon, lead, zinc, nickel) indicated an exogenous origin, hypothesized to be due to inhaled air pollution particles depositing in the nasal passages and translocating to the brain. In support of this hypothesis, studies in rats have demonstrated nanoparticle depositing on the nasal mucosa can translocate to the brain (12). The silicon and heavy metal particles observed in the brain were processed endogenously by ferritin coating and were associated with a localized inflammatory response. The studies on human tissues indicate nanosized particles may translocate to non-respiratory tissues and trigger a localized tissue response. The differences between the animal studies described by Creutzenberg et al. and that observed in humans in extra-pulmonary particle translocation may reflect the sensitivity of the detection methodologies used.

The potential for nanoparticles to elicit effects on DNA and in non-pulmonary tissues was addressed in presentations by Drs. Moller, Schins, Hougaard, and McCunney.

Dr. Moller discussed research on multiwalled carbon nanotubes (MWCNT) reporting MWCNT-7 (a long, straight, thick fiber) elicits inflammation and production of reactive oxygen species when injected into peritoneal cavities of rats; however, no DNA damage was detected. Comparable results were observed with NM-401, another long, thin nanotube, however, intraperitoneal injection of shorter nanotubes did not produce inflammatory effects. *In vitro*, MWCNT-7 nanotubes produce DNA damage, albeit only at extremely high doses. These findings on material dependent differences in inflammatory and DNA damaging effects are consistent with other studies demonstrating differences in bioactivity of various MWCNT (13) and support the concept the composition, in addition to size and shape are key to nanomaterial toxicity. Dr. Moller noted a key gap in our understanding of nanotube genotoxicity is the absence of data indicating whether any the DNA damage detected was irreversible and produced mutation.

Regarding the potential for neurotoxic effects, Dr. Schins reported inhalation of diesel exhaust increases b amyloid protein accumulation in the brains of 5 × FAD mice, a model of Alzheimer's Disease amyloid protein accumulation. These findings suggest inhaled diesel exhaust can have neurological effects distance from the respiratory tract. Additional studies on inhaled engineered nanoparticles (ZrO_2_, CeO_2_) did not increase brain plaque formation in 5 × FAD mice and were without effect in a mouse model of atherosclerosis. Studies on oral exposure of mice to titanium or silver nanoparticles had no effect on markers of neuroinflammation with some behavioral changes observed in female mice as well as biochemical changes in cortical tyrosine kinase activity. Overall, the neurotoxicity research summarized supports that inhaled or ingested nanoparticles may have effects distance from the lungs or GI tract with the effects being material dependent.

Dr. Hougaard reviewed the potential developmental and reproductive effects of nanoparticles investigating genotoxic, nervous system, reproductive and immune system changes in offspring of pregnant mice exposure to titanium dioxide, diesel particles, carbon black, and multiwalled carbon nanotubes (MWCNT). The studies supported exposure-associated changes on the central nervous system, immune system, and male fertility. The mechanisms underlying the changes reported by Drs. Schins and Hougaard were not defined and will require further investigation. The association of occupational carbon black exposure with cardiovascular disease was discussed by McCunney et al.. Environmental exposure to PM 2.5 particles has been associated with cardiovascular disease, a relationship considered to be causal in nature, although the mechanisms are unclear (8, 9, 14). Carbon black is >98 carbon and can have polycyclic aromatic hydrocarbons tightly bound to its surface. In the occupational setting, carbon black typically exists as agglomerates of nanosized particles.

McCunney et al. reviewed the three major occupational epidemiology studies on carbon black investigating populations in the United States, Great Britain, and Germany which collectively have includes over 9,300 workers. After accounting for cigarette smoking, none of these studies have demonstrated an increased risk of mortality or cardiovascular disease. A meta-analysis of these studies confirmed an absence of an association between occupational carbon black exposure and heart disease, ischemic heart disease, or acute myocardial infarction. The negative studies on carbon black considered alongside the evidence on air pollution and cardiovascular disease suggest the complex chemical properties of air pollution are a critical factor in the elevated cardiovascular disease risk vs. a specific effect of poorly soluble nanosized particles (15).

To support the development and risk assessment of nanotechnologies, the “Gracious 2020 Project” established a framework to apply grouping and read-across approaches (16). The Gracious framework describes a hypothesis- driven approach for leveraging existing data to streamline safety evaluation in terms of time, cost, and animal usage safety. The framework defines criteria for grouping, selection of appropriate benchmark materials and developing an integrated approach to testing and assessment (IATA). The Gracious framework supports both qualitative and quantitative risk assessments and, when needed, developing an appropriate experimental plan to address data gaps using *in silico, in vitro*, and/or *in vivo* testing. A challenge in using this approach may be the paucity of robust inhalation toxicology data on nanomaterials.

## Science and regulation

The aim of this final session was to explore how the role of sound and evidence- based scientific underpinning was used in regulatory standard considerations regarding PSLTs and other particles. Although regulatory concern regarding PSLTs has focused on lung cancer risk as a result of rat inhalation overload studies, this conference also addressed all relevant health end points, including respiratory tract and lung inflammatory changes, genetic and reproductive issues, among others, consistent with ECHA and other national and international guidelines. These issues relate to both hazard classification and to risk-based exposure limit setting.

The first-scene setting presentation addressed the regulatory application of science and stakeholder engagement in the setting of EU occupational exposure limits (OELs) by Alick Morris from the EU Health and Safety at Work Unit, DG Employment, Social Affairs and Inclusion, European Commission, Luxembourg. His presentation described how this activity takes place at the EU level when setting limit values (OELs) to protect the health of workers from risks due to occupational exposure to chemicals. Alick Morris described the EU Pillar of Social Rights Action Plan, the EU Occupational Safety and Health strategic framework and how the key steps in setting OELs and Biological Limit Values (BLVs) as well as their benefits. He noted how the social partners (industry, trade unions, and EU Member State governmental representatives) participate in regulatory decision-making through the EU Advisory Committee on Safety and Health (ACSH) which also adopts new lists of priorities for future limit values. He also described some current initiatives driven by the Occupational Safety and Health Directive and the Carcinogens and Mutagens Directive which included recent work on asbestos, lead and diisocyanates. He pointed out how this work was assisted *via* expert committees, formerly though the Scientific Committee on Exposure Limits (SCOEL) but more recently, by the EU European Chemical Agency's (ECHA's) Risk Assessment Committee (RAC) (https://ec.europa.eu/social/main.jsp?catId=148&langId=en).

Schulte et al. from the US National Institute for Safety and Health (NIOSH) described the application of translational science approaches to protect workers exposed to nanomaterials. In his presentation he noted that nanotechnology, like translational science, is a relatively new and transdisciplinary field. Translational science in occupational safety and health (OSH) focuses on the process of taking scientific knowledge for the protection of workers from the laboratory to the workplace and back again. Translational science has been conceptualized as having number of multiple phases of research along a continuum, from scientific discovery (T0) to efficacy (T1), to effectiveness (T2), to dissemination and implementation (D&I) (T3), to outcomes and effectiveness research in populations (T4). The translational research process applied to occupational exposure to nanomaterials might well-involve similar phases. This includes basic research (T1) in the areas of toxicology, epidemiology, industrial hygiene, medicine, and engineering. He described iterative interrelationship of these four phases in some detail, including potential barriers to the implementation of solutions. Of critical importance, and in accord with the first presentation by Alick Morris, he emphasized that stakeholder participation and engagement was critical to all four phases of the translational continuum.

The next series of presentations dealt with one particulate nanomaterial; synthetic amorphous silica (SAS) to illustrate a number of scientific, ethical, and regulatory issues. In the case of many PSLTs and nanomaterials, including nanostructured SAS, the most important exposure pathway for such materials is inhalation; depending on the possible applications of the substance in foodstuffs, cosmetics, among others that dermal and oral exposure must also be taken into account. Additionally, to reduce the need for animal experiments, *in vitro* test methods are urgently needed to compare different materials, including SAS, and to test their toxicological properties for oral and inhalative exposure.

In the first of these illustrative presentations on SAS, Peter Wick described an oral *in vitro* screening of nanomaterials with an advanced *in vitro* intestine model.

Nanostructured food processing agents, which are added to prevent caking, to improve flowing or change texture of the food, might be ingested. Regulatory authorities, as well as consumers, are concerned about potential adverse effects of nano-sized materials both in food and on public health. Considering the high oral exposure of all these food additives, a better understanding of the uptake, accumulation, and biological effects of food relevant nano-sized materials at the intestinal epithelium is needed. The presentation described 10 distinctly different SAS materials with different surface areas, structures, sizes, and surface charges that had been characterized. Their biological impact was screened in Caco-2 cell line, representative of the most common cell type in the intestine. No acute impairment of viability or barrier integrity was identified. In the second part of this work, an advanced co-culture model was established to better evaluate the impact of food grade materials in an *in vivo* like setting. The exposition of the advanced coculture model with six different SASs selected due to the different production routes, specific surface areas and different silanol content led to no differences in the viability, barrier integrity, microvilli function, and lipid uptake. Nevertheless, the treatment had shown that the mucus production increases after the treatment with SAS materials that present certain aggregate sizes and a high silanol content. A co-effect has been found for the investigation of the iron uptake. Precipitated SAS with a small specific surface area decreased the iron uptake in the advanced co-culture only in iron uptake but not in the also on the gene level. The results show that the use of this advanced *in vitro* model could lead to an improved prediction on potential adverse outcomes of food components on the intestine. Mucus seems to be a very important protective barrier in the interaction of food components with the intestinal epithelium and should be studied in more detail. The advanced co-culture model established in this work can be used for an initial estimate of the interactions of food components with intestinal epithelium and ideally, a further reduction of animal experiments in the future. Overall, this *in vitro* test model for oral intake showed that no adverse effects were observed with SAS. Further information on this alternative screening method for oral exposure is provided by Hempt et al. (17, 18).

Wiemann et al. addressed animal welfare aspects in his presentation: “Can we reduce animal testing- tiered approach using *in vitro* screening?” He presented a well-established macrophage *in vitro* method for comparative studies after inhalation of nanomaterials, which allows a grouping of the substances with regard to their activity in a serum-free test system (19, 20). Serum lowers the bioactivity in this macrophage *in vitro* test system. The influence of serum is described in a separate publication by Wiemann et al. (21). He presented the results of SAS that showed that bioactivity *in vitro* is strongly diminished by protein binding to the particle surface. Of special interest in the investigation was the bioactivity of SAS surface-treated with organosilanes in comparison with non-treated SAS forms using alveolar macrophages as a highly sensitive test system (Wiemann et al.). Five non-treated and nine surface-treated SAS (one hydrophilic, eight hydrophobic, six different coating reagents) were included in the *in vitro* study with alveolar macrophages. Dispersion of the hydrophobic SAS (8/14) required pre-wetting with ethanol and extensive ultrasonic treatment in the presence of 0.05% BSA (Protocol 1). Hydrophilic SAS were suspended by moderate ultrasonic treatment (Protocol 2) and with Protocol 1.

Importantly, the results of these *in vitro* studies correlate very well with the results of subchronic *in vivo* studies (90-day study) with hydrophobic surface-treated SAS (22). In this study, hydrophobic surface-treated SAS showed a weaker inflammatory activity at the end of exposure and faster reversibility of effects in the recovery period compared to pure pyrogenic SAS.

The results above raise the question that if hydrophobic surface-treated SAS materials show reduced bioactivity *in vitro* and in long-term *in vivo* studies, the question arises as to why lethality can be observed in some acute inhalation studies with very high concentrations of hydrophobic surface treated SAS. The answer to this question on lethality was addressed by Jürgen Nolde and Nils Krueger. “The challenge to create particulate aerosols for acute toxicity testing—a systematic approach” was presented by Juergen Nolde. Large differences between lethal and non-lethal concentrations of different forms of the same substance have been documented through a large number of acute inhalation studies which do not conform to the results from acute oral or dermal studies which did not provide any concerns on potential toxicity for the same substance. Therefore, it is necessary to address the cause of these contrary results in the different behaviors of the particles of the substance in the inhalation equipment, up to the point when the particles are delivered to the nose of the rat. OECD test guidelines for acute inhalation studies require defined maximum particle sizes (MMAD max. 4 μm) and concentrations up to 5,000 mg/m^3^ or the maximum technical feasible concentration. This technical feasible concentration, however, and more importantly, its measurement, is not defined. The challenge of creating particulate aerosols for acute toxicity testing using a systematic approach was presented. The aim is to examine optimized aerosol generation and its monitoring, including detailed characterization of the exposure atmospheres in the test equipment (stability, particle concentration, particle size distribution over time) from the point of generation to the point of release out of the system prior to performing OECD animal inhalation studies (Wessely et al.). It was the intention to perform rat studies with the highest technically feasible concentration without significant aerosol altering. Studies of aerosol generation on this scale and detail with several different particular substances, e.g., silica, organic pigments, aluminum oxide, sugar, flour dust, and calcium carbonate have not previously been carried out in any acute animal inhalation studies. A key conclusion was that a detailed evaluation of the aerosol generation can help predict the outcome of rat inhalation studies with particles and therefore, reduce the number of animals required in future acute inhalation tests.

Krueger et al. presented a study on hydrophobic surface treated SAS: “Non-specific particle effects now trigger classification?.” The aim of the study was to understand the mechanism of lethality associated with high dose inhalation of hydrophobic surface treated SAS observed in some acute inhalation studies. It was demonstrated that physical obstruction of the upper respiratory tract (nasal cavities) caused effects observed when hydrophobic surface-treated SAS was inhaled (flow-past, nose-only) by six Wistar rats (three males and three females) in an acute toxicity study at a concentration of ~500 mg/m3 for 4 h (Krueger et al.). Under the conditions of the test set-up, the concentration applied was found to be the highest that can be delivered to the test animal port without significant alteration of the aerosol size distribution over time. None of the test-material-exposed animals survived the observation period. Histopathology and energy dispersive X-ray (EDX) analysis demonstrated that test material particles agglomerated and formed a gel-like substrate that blocked the upper respiratory airways, which is fatal for the rat as an obligatory nose breather. This observation is in line with the findings reported by Hofmann et al. (23) showing a correlation between lethality and hydrophobicity determined by contact angle measurement.

## Summary

This 2 day conference with 30 speakers from Europe and the USA addressed the scientific basis of potential health risks associated with exposure to poorly soluble low toxicity particles (PSLTs), most notably carbon black, titanium dioxide and talc. From this conference, 20 peer reviewed scientific papers were published in this special issue. The role of epidemiology and toxicology in assessing potential human health risks of regulatory impact was emphasized. Clearly, as regulatory challenges persist in establishing appropriate classification schemes and exposure limits for PSLTs, the role of scientific investigations will play a major role.

## Author contributions

All authors listed have made a substantial, direct, and intellectual contribution to the work and approved it for publication.

## References

[1] Sorahan TL Hamilton M van Tongeren K Gardiner Harrington JM. A cohort mortality study of U.K. carbon black workers, 1951–1996. Am J Ind Med. (2001) 39:158–70. 10.1002/1097-0274(200102)39:2<158::aid-ajim1003>3.0.co;2-l 11170158

[2] Wellmann J Weilland SK Neiteler G Klein G Straif K. Cancer mortality in German carbon black workers 1976–98. Occup Environ Med. (2006) 63:513–21. 10.1136/oem.2006.026526 16497850 PMC2078133

[3] Dell LD Gallagher AE Crawford L Jones RM Mundt KA. Cohort study of carbon black exposure and risk of malignant and nonmalignant respiratory disease mortality in the US carbon black industry. J Occup Environ Med. (2015) 57:984–97. 10.1097/JOM.0000000000000511 26340287 PMC4556099

[4] Ward EM Schulte PA Straif K Hopf NB Caldwell JC Carréon T. Research recommendations for selected IARC classified agents. Environ Health Perspect. (2010) 118:1355–62. 20562050 10.1289/ehp.0901828 PMC2957912

[5] Yong M Anderle L Levy L McCunney RJ. Carbon black and lung cancer mortality-a meta-regression analysis based on three occupational cohort studies. J Occup Environ Med. (2019) 61:949. 31513042 10.1097/JOM.0000000000001713

[6] Dweik RA Boggs P Erzurum SC Irvin CG Leigh MW Lundberg JO. An official ATS clinical practice guideline. AJRCCM. (2011) 184:602–15. 10.1164/rccm.9120-11ST 21885636 PMC4408724

[7] Kazani S Planaguma A Ono E Bonini M Zahid M Marigowda G. Exhaled breath condensat eicosanoid levels associate with asthma and its severity. J Allergy Clin Immunol. (2013) 132:547–753. 10.1016/j.jaci.2013.01.058 23608729 PMC3759610

[8] Pope CA Burnett RT Thurston GD Thun MJ Calle EE Krewski D. Cardiovascular mortality and long-term exposure to particulate air pollution: epidemiological evidence of general pathophysiological pathways of disease. Circulation. (2004) 109:71–7. 10.1161/01.CIR.0000108927.80044.7F 14676145

[9] Hayes RB Lim C Zhang Y Cromar K Shao Y Reynolds HR. P < 2.5 air pollution and cause-specific cardiovascular disease mortality. Inter J Epidemiol. (2020) 49:25–35. 10.1093/ije/dyz114 31289812 PMC7124502

[10] Hougaard KS Campagnolo L Chavette-Palmer P Tarrade A Rousseau-Ralliard D Valentino S. A perspective on the developmental toxicity of inhaled nanoparticles. Reprod Toxicol. (2015) 56:118–40. 10.1016/j.reprotox.2015.05.015 26050605

[11] Miller MR McLean SG Duffin R Lawal AO Araujo JA Shaw CA. Diesel exhaust particulate increases the size and complexity of lesions in atherosclerotic mice. Part Fibre Toxicol. (2013) 10:61. 10.1186/1743-8977-10-61 24330719 PMC3907045

[12] Oberdörster G Sharp Z Atudorei V Elder A Gelein R Kreyling W. Translocation of inhaled ultrafine particles to the brain. Inhal Toxicol. (2004) 16:437–45. 10.1080/08958370490439597 15204759

[13] Moller P Wils RS Ianni ED Guterierrez CAT Rousgaard M Jaconsen NR. Genotoxicity of multiwalled carbon nanotube reference materials in mammalian cells and animals. Mutat Res. (2021) 288:108393. 10.1016/j.mrrev.2021.108393 34893158

[14] Shin HH Fann N Burnett RT Cohen A Hubbell BJ. Outdoor fine particles and nonfatal strokes: systematic review and metaanalysis. Epidemiology. (2014) 25:835–42. 10.1097/EDE.0000000000000162 25188557 PMC4222795

[15] Slawsky E Ward-Caviness CK Neas L Devlin RB Cascio W Russell AG. Evaluation of PM2.5 air pollution sources and cardiovascular health. Environ Epidemiol. (2021) 5:e157. 10.1097/EE9.0000000000000157 34131618 PMC8196100

[16] Stone V Gottardo S Bleeker EAJ Braakhuis H Dekkers S Fernandes T. A framework for grouping read-across of nanomaterials-supporting innovation and risk assessment. Nanotoday. (2020) 35:100941. 10.1016/j.nantod.2020.100941

[17] Hempt C Kaiser JP Scholder O Buerki-Thurnherr T Hofmann H Rippl A. The impact of synthetic amorphous silica (E 551) on differentiated Caco-2 cells, a model for the human intestinal epithelium. Toxicol In Vitro. (2020) 67:104903. 10.1016/j.tiv.2020.104903 32473318

[18] Hempt C Cordula H Hannig Y Rippl A Wick P Buerki-Thurnherr T. Investigating the effects of differently produced synthetic amorphous silica (E 551) on the integrity and functionality of the human intestinal barrier using an advanced *in vitro* co-culture model. Arch Toxicol. (2021) 95:837–52. 10.1007/s00204-020-02957-2 33319326 PMC7904742

[19] Wiemann M Vennemann A Sauer UG Wiench K Ma-Hock L Landsiedel R. An *in vitro* alveolar macrophage assay for predicting the short-term inhalation toxicity of nanomaterials. J Nanobiotechnol. (2016) 14:16. 10.1186/s12951-016-0164-2 26944705 PMC4779246

[20] Koltermann-Jülly J Johannes G Antje K Kai Werle V Müller P Robert L. Abiotic dissolution rates of 24 (nano)forms of 6 substances compared to macrophage-assisted dissolution and *in vivo* pulmonary clearance: grouping by biodissolution and transformation. NanoImpact. (2018) 12:29–41. 10.1016/j.impact.2018.08.005

[21] Wiemann M Vennemann A Venzago C Lindner G-G Schuster TB Krueger N. Serum lowers bioactivity and uptake of synthetic amorphous silica by alveolar macrophages in a particle specific manner. Nanomaterials. (2021) 11:628. 10.3390/nano11030628 33802450 PMC7999370

[22] Reuzel PG Bruijntjes JP Feron VJ Woutersen RA. Subchronic inhalation toxicity of amorphous silicas and quartz dust in rats. Food Chem Toxicol. (1991) 29:341–54. 10.1016/0278-6915(91)90205-L 1648030

[23] Hofmann T Ma-Hock L Teubner W Athas J-C Neubauer N Wohlleben W. Reduction of acute inhalation toxicity testing in rats: the contact angle of organic pigments predicts their suffocation potential. Appl In Vitro Tox. (2018) 4:220–8. 10.1089/aivt.2018.0006

[24] Davis MD Montpetit A Hunt J. Exhaled breath condensate: an overview. Immmunol Allergy Clin North Am. (2012) 32:363–75. 10.1016/j.iac.2012.06.014 22877615 PMC3417047

